# Fairness in ambulance routing for post disaster management

**DOI:** 10.1007/s10100-021-00785-y

**Published:** 2021-10-26

**Authors:** Roberto Aringhieri, Sara Bigharaz, Davide Duma, Alberto Guastalla

**Affiliations:** 1 Dipartimento di Informatica, Università degli Studi di Torino, Corso Svizzera 185, 10149 Turin, Italy; 2 Department of Industrial Economics and Technology Management, Faculty of Economics and Management, NTNU, 7491 Trondheim, Norway; 3 Dipartimento di Matematica “Felice Casorati”, Università degli Studi di Pavia, via Adolfo Ferrata, 5, 27100 Pavia, Italy

**Keywords:** Post disaster management, Ambulance routing, Fairness, Team orienteering problem

## Abstract

Disaster management generally includes the post-disaster stage, which consists of the actions taken in response to the disaster damages. These actions include the employment of emergency plans and assigned resources to (i) rescue affected people immediately, (ii) deliver personnel, medical care and equipment to the disaster area, and (iii) aid to prevent the infrastructural and environmental losses. In the response phase, humanitarian logistics directly influence the efficiency of the relief operation. Ambulances routing problem is defined as employing the optimisation tools to manage the flow of ambulances for finding the best ambulance tours to transport the injured to hospitals. Researchers pointed out the importance of equity and fairness in humanitarian relief services: managing the operations of ambulances in the immediate aftermath of a disaster must be done impartially and efficiently to rescue affected people with different priority in accordance with the restrictions. Our research aim is to find the best ambulance tours to transport the patients during a disaster in relief operations while considering fairness and equity to deliver services to patients in balance. The problem is formulated as a new variant of the team orienteering problem with hierarchical objectives to address also the efficiency issue. Due to the limitation of solving the proposed model using a general-purpose solver, we propose a new hybrid algorithm based on a machine learning and neighbourhood search. Based on a new set of realistic benchmark instances, our quantitative analysis proves that our algorithm is capable to largely reduce the solution running time especially when the complexity of the problem increases. Further, a comparison between the fair solution and the system optimum solution is also provided.

## Introduction

The destructive impacts of disasters have threatened societies for years and inevitable consequences entail financial and human costs for the victims. However, today’s accurate, effective and quick responses by decision-makers can reduce the damages and loss of human life. The performance of humanitarian organisations against the natural disasters such as earthquake, flood and storm, and human causes such as fire, environmental pollution and war should be measured in several dimensions to optimise the speed of operations and offer high flexibility services as well as cost minimisation’s (Vargas Florez et al. 2015). To achieve that, disaster management by emphasising planning, prioritisation and decision making is a necessity in relief operations (Talarico et al. 2015).

Disaster management generally includes pre- and post-disaster stages. Pre-disaster stage predicts the potential human and property losses and develops the preparedness plans to reduce the impact of disasters by improvement in emergency services and humanitarian logistics while the post-disaster stage consists of the actions taken in response to the disaster damages (Shiripour and Mahdavi-Amiri 2019). These two phases have been classified into four action categories in more detail: (i) mitigation refers to the actions needed to prevent the occurrence of a disaster and to decrease the disastrous impacts; (ii) preparedness involves planning procedures in a community for a timely response to damages; (iii) response includes the employment of emergency plans and assigned resources to (a) rescue affected people immediately, (b) deliver personnel, medical care and equipment to the disaster area, and (c) aid to prevent the infrastructural and environmental losses; (iv) recovery is the final action category in which actions followed to return the situation to normalcy (Boonmee et al. 2017; Altay and Green 2006).

In the response phase, humanitarian logistics directly influence the efficiency of the relief operation. Properly planned transportation of relief supplies and medical care to a disaster area by ambulances prevent human losses (Ahmadi et al. 2015). Hence, it is necessary to employ optimisation models to address the challenge of assistance to the wounded. The ambulances routing problem is defined as employing the optimisation tools to manage the flow of ambulances for finding the ambulance tours to transport the injured to hospitals (Tlili et al. 2018). The task of ambulances is to deliver personnel to the disaster area to assist the slightly injured and transport the seriously injured to hospital or shelter. The lack of medical services in disaster situations increases the possibility of death and human suffering. To address, it is crucial to properly include the concept of fairness in emergency relief routing optimisation for injured victims (Zhu et al. 2019).

Although many researchers concerning humanitarian relief services pointed out the importance of equity, the few articles focused on fairness as an objective in disaster optimisation problems. Modelling fairness is in itself challenging that resulted in modelling approaches determining solutions with different quality. The concept of fairness has been varied in definition in accordance with to the context of problems. Regarding relief operations, fairness can be defined as equity and impartiality at the service level for people who are in need (Anaya-Arenas et al. 2018; Huang et al. 2012). Hence, managing the operations of ambulances in the immediate aftermath of a disaster must be done impartially and efficiently to rescue affected people with different priority in accordance with the restrictions.

Our research aim is to find the best ambulance tours to transport the patients in relief operations after a disaster while considering fairness and equity to deliver services to patients in balance. The problem is formulated as a new variant of the Team Orienteering Problem (TOP) (Butt and Cavalier 1994; Chao et al. 1996) with hierarchical objectives to address also the efficiency issue. Due to the limitation of solving the proposed model using a general-purpose solver as it will be shown in our quantitative analysis, we propose a new hybrid algorithm based on a machine learning and neighbourhood search. Based on a new set of realistic benchmark instances, our quantitative analysis proves that our algorithm is capable of largely reducing the solution running time especially when the complexity of the problem increases. Further, a comparison between the fair solution and the system optimum solution is provided in order to evaluate the so called price of fairness (Nicosia et al. 2017).

The paper is organised as follows. We review the most relevant researches in the literature in Sect. 2 also providing an introduction to the TOP highlighting the new characteristics of our problem with respect to the current TOP literature. The ambulance routing problem is described and modelled in Sect. 3. The hybrid algorithm is described in Sect. 4. The quantitative analysis based on the set of realistic instances is reported and discussed in Sect. 5. Conclusions, challenges and future works are discussed in Sect. 6.

## Literature review

Emergency logistics have motivated many researchers to address humanitarian aid problems. As can be seen in surveys (Anaya-Arenas et al. 2014; Hoyos et al. 2015; Ozdamar and Ertem 2015) disaster management is a challenging area to investigate. Among the disaster management problems, disaster relief routing is considered of the utmost importance in which researchers attempt to optimise the relief aid transportation in post-disaster situations. In this context, Nolz et al. (2011) formulated a multi-objective optimisation problem to deliver the relief supplies to casualties. The objectives considered are to minimise the risks and total travel time and to maximise the population coverage of relief commodities. Ahmadi et al. (2015) proposed a multi-depot location routing model considering failure in the transportation network. The model determined the locations of local depots and routing for last-mile distribution whereas the network destruction was modelled via a two-stage stochastic problem and solved by a neighbourhood search algorithm. Sayyady and Eksioglu (2010) proposed a mathematical model to find the best evacuation routes for transit vehicles to minimise the number of casualties and the total evacuation time. A few articles considered multi-commodity network flow models to integrate transportation of relief commodities and injured people such as Yi and Ozdamar (2007) and Najafi et al. (2013). Sabouhi et al. (2019) developed a routing and scheduling model for people evacuation from affected areas to shelters and distribution of relief commodities while considering the minimisation of the sum of arrival times of vehicles at affected areas, shelters, and distribution centres.

The distribution of humanitarian aids (inbound logistics) such as water, food, shelters and medicine to the casualties are only part of the relief operation while providing medical services to help the injured and the transportation of patients to hospitals (outbound logistics) are other issues that have been focused on in several papers. For instance, Chiu and Zheng (2007) integrated mobilisation destination, traffic assignment, and departure schedule concepts to formulate a linear program to dynamically model multi-priority groups of patients. Ozdamar and Demir (2012) suggested a hierarchical cluster and route procedure for dealing with a disaster relief commodities delivery and casualty pick up problem. The aim was to optimise the allocation of warehouses and hospitals to clustering demand centres. Talarico et al. (2015) developed two ambulance routing models for disaster response management where patients are divided into two groups: low-priority injured people and high-priority patients which should be transferred to the hospital. The model minimises the sum of the weighted maximum service completion time for low-priority and high-priority patients. Tlili et al. (2017) proposed a vehicle routing problem (VRP) with pickup and delivery to model the route of the ambulances when a disaster occurs.

Scarce resources imposed restrictions in assistance to the victims and it has always been highly problematic to deliver relief supplies and medical services in an equal manner. Fairness approaches used in relief distribution as an objective can be categorised into three main groups (Anaya-Arenas et al. 2018).

The first type is based on the Rawlisan approach in which the focus is on maximising the minimum outcome (Karsu and Morton 2015). For instance, Tzeng et al. (2007) presented a multi-period multi-objective relief distribution model to minimise the total costs and total travel times as well as maximising the minimal satisfaction at each period. Sun et al. (2014) developed a multi-objective patient allocation model to minimise the maximum distance a patient travels to a hospital in a pandemic outbreak.

The second approach deals with providing fairness recognised as a deviation measure. Lin et al. (2011) introduced a multi-items, multi-vehicles, multi-periods model to minimise the range of the unsatisfied demands. Huang et al. (2012) measured the effect of efficiency, efficacy, and equity on the structure of vehicle routes and the distribution of resources.

Deprivation approach is the third one, which is less used due to its difficulty. Holguín-Veras et al. (2012) proposed social costs as an objective function for the post-disaster humanitarian logistics problem. Social costs can be achieved through logistic costs and deprivation costs defined as the economic valuation of suffering experienced by people in catastrophic events.

The fairness approach in the transportation of injured victims was used by Zhu et al. (2019). The authors suggested a relative deprivation cost as an objective to provide equity. The problem was solved by an ant colony meta-heuristic algorithm and compared with a genetic algorithm. The integration of ambulance routing problem and fairness approach is a research area which should be further emphasised considering also the price of fairness, a quality measure introduced by Nicosia et al. (2017), which is a comparison between the system optimum solution and a fair solution.

Our problem is positioned in the context of the outbound logistics and, more precisely, in the ambulance routing problem framework, which consists in finding the ambulance tours to transport the injured people to hospitals (Tlili et al. 2018). Our research is inspired by the work reported in Talarico et al. (2015). Our problem is modelled in Sect. 3 as a variant of the TOP (see Vansteenwegen et al. 2011; Gunawan et al. 2016; Vansteenwegen and Gunawan 2019 for more detailed literature reviews), which is a routing problem with profits and multiple vehicles. The class of the Vehicle Routing Problems with Profits (VRPPs) is characterised by the fact that not all customers can be served. This implies the need to consider two different decisions as reported in Archetti et al. (2014), that is (i) which customers to serve, and (ii) how to cluster the customers to be served in different routes (if more than one) and order the visits in each route. The customer selection is driven by a profit associated with each customer that makes such a customer more or less attractive.

Erdogan and Laporte (2013) introduces the Orienteering Problem with Variable Profits (OPVP) in which a single vehicle can collect the whole profit at the customer after a discrete number of “passes” or spending a continuous amount of time. As in the classical orienteering problem, the objective is to determine a maximal profit tour for the vehicle, starting and ending at the depot, and not exceeding a travel time limit.

Angelica Salazar-Aguilar et al. (2014) introduce an extension of the TOP by considering the multi-district aspect, a set of mandatory and optional tasks located in several districts and some incompatible tasks which cannot be carried out during the same day. The problem is called Multi-District TOP (MDTOP). It is required to perform all mandatory tasks over the planning horizon, while the optional tasks are only executed if time permits.

Our problem has service times at nodes as in Erdogan and Laporte (2013) and mandatory and optional nodes as in Angelica Salazar-Aguilar et al. (2014). To the best of our knowledge, this is the first research attempt to consider both aspects at the same time. Further, in accordance with the literature reviews currently available, this is the first application of the TOP to the case of humanitarian logistics.

## Problem statement and mathematical formulation

The organisation leading the answer to a disaster differs country by country, and it usually depends on different history, characteristics and needs of each different national civil security systems (see, e.g., Boin et al. 2014). For instance, the Italian civil security systems is based on the *subsidiarity principle* (Di Camillo et al. 2014), which means that as soon as the calamitous event becomes larger in terms of geographical distribution and/or intensity, different and larger administration are involved. On the contrary, in other EU countries the system is more centralised. These differences determine different organisation models at the tactical and operational level. In this paper, the following problem statement is inspired by that reported in Talarico et al. (2015).

When a disaster occurs, initial data about the damages and injuries is collected as fast as possible. The dispatcher classifies patients’ requests according to their severity and locations. The ambulances staffed by medical crew are dispatched to affected areas immediately to treat wounded people and transport patients to hospitals as needed. Two groups of patients based on a triage system can be considered in the affected area. Red patients must be transported to hospitals because they suffer from serious injuries. On the contrary, green patients are people who are slightly injured and, by consequence, need only a first aid directly on the field.

Priority scores for green patients are introduced to define which of them is less or more urgent, that is a preference about who should be rescued in a short time. Therefore, all green patients have a score that can be interpreted as urgency level weights, which are used to lead the selection of the most urgent green patients during the optimisation. Maximising the overall score for green patients can be considered as a goal to provide an efficient service. As the work-shift duration can provide, for job safety reasons, a time limitation for each ambulance, all the green patients may not be visited, and those with the higher scores should have the priority. On the other hand, red patients are suffering from serious injuries and should be transferred to hospitals to be cured with adequate medical equipment. Minimising the maximum waiting time for red patients can be considered as a goal to provide equal services.

To satisfy the needs of patients, an ambulance starts its tour from a depot (e.g., hospitals or medical centres) to visit patients in affected areas and returns to a hospital. The personnel of the ambulance treat a green patient in the field and visit another green patient in its tour while after visiting the red patient, the ambulance has to pick the patient and take her/him to one of the available hospitals in the area, not necessarily the closest one. In our operative context, we assume that the hospitals have enough capacity to treat all the patients: actually, a common practice is that patients are stabilised and then transferred to other hospitals to free up space and resources.

The ambulance routing problem based on the TOP can be formulated to find the optimal tour for ambulances to deliver the services to patients after a disaster. Note that in the TOP each node can be visited once except for the source node and the destination node. Then, we can define a network on a completed graph $E = (N, A)$, where *N* is the set of nodes representing locations over the considered area and $A = \{(i, j): i,j \in N\}$ is the set of arcs indicating the connections between each pair of such locations. Three main types of nodes are considered in this problem: (i) green patients (set *G*), (ii) red patients (set *R*), and (iii) actual hospitals (set *O*). To this types of node, we add several dummy nodes in order to bring the problem in the TOP formulation, that is: (iv) the dummy source and destination depot nodes 1 and *n*, and (v) several dummy hospitals (set *D*), which are replications of the actual hospital nodes (i.e. they have same coordinates of the original nodes of the set *O*) that allows us to visit two or more times the same actual hospital visiting two different nodes. The number of replications of each actual hospital node is computed in such a way to allow the visit of such nodes as a starting hospital for the assigned ambulances and for the medical care of all the red patients (worst case). Furthermore, we indicate with $P = G \cup R$ the set of all patients and with $H = O \cup D$ the set of all (actual and dummy) hospitals. Therefore, $N = P \cup H \cup \{1, n\}$. We set $n=|N|$ and we enumerate all nodes from 1 to *n*. The priority score of green patients is denoted by $s_i$, while the set of available ambulances is indicated with *K*.

**Fig. 1:**
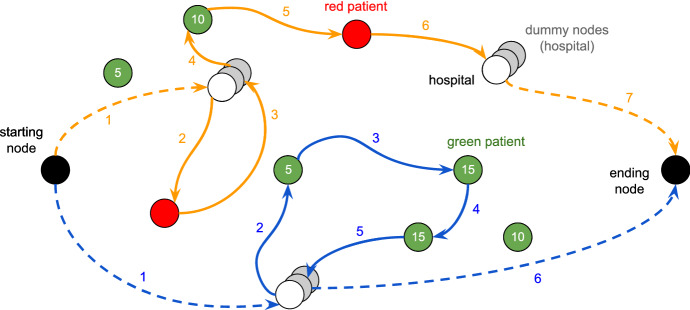
Example: solution of a TOP formulation representing the ambulance tours after a disaster. Each tour starts from the starting dummy node and ends in the ending dummy node, and hospitals are always visited in accordance with the graphic overlay. Numbers on arcs indicate the order in which they are visited in the tour

An example of the introduced graph and the related solution is explained in Figure 1. Nodes of sets *G*, *R* and *O* are coloured in green, red and white, respectively. Labels on green nodes indicate the scores $s_i, i \in G$. In correspondence of each node of *O*, several replications are provided in order to suit our problem to the TOP framework; such nodes, in grey, are the elements of *D*. Black nodes represent the dummy depots $\{1,n\}$. Two ambulance tours are represented by the sequence of coloured arcs (orange and blue) from the source depot to the destination depot: continuous arcs indicate the actual moving of the ambulance between two physical places represented by the nodes, while dashed arcs are only used to connect the dummy depots to the actual hospital where the ambulance is located at the beginning and at the end.

The connections of the node 1 are set in accordance with the instance of the problem: the first arc visited by the tour of an ambulance is that between 1 and one of the nodes related to the hospital in which it actually is located at the beginning. Such an initial location is given by the parameter $l_{hk}$, which is equal to 1 if the ambulance $k \in K$ is initially parked at the hospital *h*. The connections of the node *n* have the same meaning, but they are part of the solution because there is not any constraint about the final destination of the ambulances (except that it must be a hospital node). Furthermore, we observe that each intermediate node is visited at most once: if a hospital needs to be visited twice or more, then a different node belonging to *H*, and related to that hospital, is visited every time; for instance, the orange tour visits the actual hospital node firstly and a dummy node for the second visit. We remark that red nodes must be visited once, while green, white and grey nodes could be not visited.

Since the locations of patients and hospitals are identified, the travel time between a pair of nodes $i, j \in N$ is defined as $t_{ij}$, where:

- $$t_{ij} = t_{ji}$$ for each $$i,j \in N$$;
- $$t_{ij} = 0$$ if $$i \in \{1, n\}, j \in H$$;
- $t_{ij} = 0$ if $i, j \in H$ are replications of the same actual hospital.

We take into account also the time spent by the ambulance on the nodes, to which we will always refer as the service time $f_i$, $i \in N \setminus \{1, n\}$. Such time represents: (i) the on-place treatment duration when $i \in G$, (ii) the preparation time spent on the place when $i \in R$, and (iii) the time spent to release a red patient to the hospital when $i \in H$. We assume that hospitals can accept red patients regardless of the hospital capacity, and an ambulance can only carry one red patient at a time without giving other patients treatment in the meantime. We fix a maximum time $T_{max}$ for each ambulance to provide treatments to patients and to complete its tour moving to a hospital. We resume the notation used in the model in Table 1.

**Table 1:** Notation

Sets		Parameters	
*N*	All nodes	$t_{ij}$	Travelling time between *i* and *j*
*G*	Green patients	$f_i$	Service time in the node *i*
*R*	Red patients	$T_{max}$	Maximum tour duration
*P*	All patients	$s_i$	Priority score of the green patient *i*
*O*	Actual hospitals	$l_{hk}$	Initial location of *k* (1 if it is *h*, 0 otherwise)
*D*	Dummy hospitals		
*H*	All hospitals		
*K*	Ambulances		
**Decision variables**			
$x_{ijk}$	Takes 1 if ambulance *k* visits node *i* directly before node *j*, 0 otherwise		
$y_{ik}$	Takes 1 if ambulance *k* visits patient *i*, 0 otherwise		
$z_{hk}$	Takes 1 if ambulance *k* visits hospital *h*, 0 otherwise		
$m_{ih}$	Takes 1 if red patient *i* is transported to hospital *h*, 0 otherwise		
$u_{ik}$	Position of the patient *i* in the tour of the ambulance *k*		
$w_i$	Visit time of the node *i* (waiting time if *i* is a patient)		
$C_{max}$	Maximum completion time of all red patients		

Let us introduce the linear programming model of our problem based on the TOP framework. Constraint (1) ensures that all ambulances start their visit from the dummy depot node 1 and end on the dummy depot node *n*.

1 $$\begin{aligned} \sum _{k \in K}\sum _{j \in P} x_{1jk} = \sum _{k \in K} \sum _{i \in P} x_{ink} = |K| \end{aligned}$$

Constraints (2) impose that at the beginning each ambulance $k \in K$ is located to the hospital node $h \in H$, in accordance with the value of the parameter $l_{hk}$.

2 $$\begin{aligned} x_{1hk} = l_{hk} \, ,\qquad \forall h \in H, k \in K \end{aligned}$$

Constraints (3)–(4) guarantee that each green patient or hospital is visited at most once and each red patient must be visited once, respectively.

3 $$\begin{aligned} \sum _{k \in K} y_{ik} \le 1 \, ,\qquad \forall i \in G \cup H \end{aligned}$$

4 $$\begin{aligned} \sum _{k \in K} y_{ik} = 1 \, ,\qquad \forall i \in R \end{aligned}$$

Constraints (5) imply that each ambulance should go to a hospital after visiting a red patient node.

5 $$\begin{aligned} \sum _{j \in P}\sum _{k \in K} x_{ijk} = 0 \, ,\qquad \forall i \in R \end{aligned}$$

Constraints (6) enforce the tour connectivity: when an ambulance visits a patient, it also has to leave that location. Furthermore, they allows the consistency of the decision variables $x_{ijk}$ and the corresponding $y_{ik}$.

6 $$\begin{aligned} \sum _{j \in N \setminus \{n\}} x_{jik} = \sum _{j \in N \setminus \{1\}} x_{ijk} = y_{ik} \, ,\qquad \forall i \in P, k \in K \end{aligned}$$

Constraints 7 ensure the respect of the time limit $T_{max}$ for each ambulance tour.

7 $$\begin{aligned} \sum _{i \in P \cup H}\sum _{j \in P \cup H} t_{ijk}x_{ijk} + \sum _{i \in P} f_i y_{ik} + \sum _{h \in H} f_h z_{hk} \le T_{max} \, ,\qquad \forall k \in K \end{aligned}$$

Constraints 8 fix $z_{ihk}$ to 1 when the hospital *h* is visited by the ambulance *k*.

8 $$\begin{aligned} x_{ihk} \le z_{hk} \, ,\qquad \forall i \in R, h \in H, k \in K \end{aligned}$$

Constraints (9)–(11) are introduced to properly compute the visit time of the nodes, that is the waiting time for patient nodes and the arrival time to the hospital for the (actual and dummy) hospital nodes. The waiting time of each patient visited directly after a green patient is fixed by the constraints (9), which take into account the waiting time and the service time of that patient, adding the travelling time between the two nodes. Similarly, constraints (10) set the waiting time of a patient visited directly after a hospital. In this case the service time at the hospital is considered only if that node is not the starting location of the ambulance. The same rationale is used for the constraints (11), which allows the computation of the arrival time at the hospital after visiting a patient node (that can be after a red patient node or after the last green patient of the ambulance tour). Big-M is used in these three constraints.

9 $$\begin{aligned} w_i + f_i + t_{ij} \le w_j + (1-x_{ijk})M , \quad&\forall i \in G, j \in P, k \in K&\end{aligned}$$

10 $$\begin{aligned} w_h + (1-l_{hk})f_h + t_hj \le w_j + (1-x_{ijk})M , \quad&\forall h \in H, j \in P, k \in K&\end{aligned}$$

11 $$\begin{aligned} w_i + f_i + t_{ih} \le w_h + (1-x_{ihk})M , \quad&\forall i \in P, h \in H, k \in K&\end{aligned}$$

**Fig. 2:**
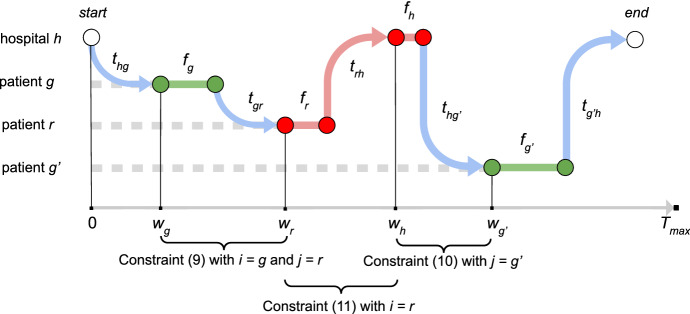
Example: how the constraints (9)- (11) work

We report an example in Fig. 2 to describe how these constraints work. In order to simplify the description, let us assume to have only one actual hospital *h* and we indicated all its dummy nodes with the same notation. Then, we consider a single tour, since the waiting times of patients visited on different tours do not affect each other. Let us consider a tour in which the ambulance starts from the hospital *h*, then it moves on the green patient *g*, which requires a certain service time and a travelling time to reach the next node, that is the patient *r*. After visiting *r* on her/his location with a duration equal to the patient’s service time $f_r$, the patient is transported to the hospital *h* (i.e., on a node which is one of its replications), where the ambulance spent other time before to travel to the next place, that is the green patient $g'$. When the service time of this patient is elapsed, the ambulance goes back to the hospital to finish the tour. Constraints (9) relate the waiting times of the patients *g* and *r*, then constraints (11) bind the visit time of *h* on the basis of $w_r$, and finally the waiting time of $g'$ is related to the visit time of *r* through constraints (10).

Constraints (12) is used to compute the maximum completion time of the red patients, that will be minimised within the proposed objective functions. The completion time is defined as the time elapsed from the beginning of the ambulance tour up to the release of the patient at the hospital. Constraints (13) are used to compute the value of the binary variable $m_{ih}$, which indicate in which hospital a red patient is transported and is used in constraints (12).

12 $$\begin{aligned} C_{max} \ge w_i + f_i + m_{ih}(t_{ih}+f_h) , \quad&\forall i \in R, h \in H&\end{aligned}$$

13 $$\begin{aligned} m_{ih} \ge \sum _{k \in K}x_{ihk} , \quad&\forall i \in R, h \in H&\end{aligned}$$

Finally, constraints (14)–(15) are necessary to prevent sub tours.

14 $$\begin{aligned} 2 \le u_{ik} \le n , \quad&\forall i \in N \setminus \{1\}, k \in K&\end{aligned}$$

15 $$\begin{aligned} u_{ik} - u_{jk} + 1 \le (n-1)(1-x_{ijk}) , \quad&\forall i,j \in N \setminus \{1\}, k \in K&\end{aligned}$$

As discussed at the beginning of this section, two objectives emerge from the analysis of the problem: the fairness in reducing the red patient waiting times, and the efficiency in collecting the green patient scores. The former is the minimisation of $C_{max}$, while the latter is the maximisation of the value $\sigma _G$ equal to the sum of the scores of all the green patients visited. To deal with these conflicting objectives at the same time, we adopt the hierarchical objective function modelling approach to represent the two objectives. Such an approach allows us to optimise the higher level objective, and then to improve as much as possible the lower level objective without deteriorating the higher level one.

We propose two hierarchical objective functions that differ in the hierarchy in order to evaluate the differences between the resulting solutions, as reported in Sect. 5. Such objective functions are subject to the constraints (1)–(15) and consist of two levels corresponding to the two different goals. The former $z_f$ (16) puts the minimisation of $C_{max}$ at the higher level and the maximisation of $\sigma _G$ at the lower level. The latter $z_e$ (17) reverses the two levels with respect to the first one. The constants $\alpha _1$ and $\alpha _2$ have to be fixed in such a way to ensure the hierarchy of the two components from a numerical point of view: we set $\alpha _1 = \sum _{i \in G}s_i + 1$ and $\alpha _2 = T_{max} + 1$.

16 $$\begin{aligned} \min \,\, z_f \,\,= & {} \,\, \alpha _1 C_{max} - \sum _{k \in K} \sum _{i \in G} s_i y_{ik} \end{aligned}$$

17 $$\begin{aligned} \max \,\, z_e \,\,= & {} \,\, \alpha _2 \sum _{k \in K} \sum _{i \in G} s_i y_{ik} - C_{max} \end{aligned}$$

As discussed in Anaya-Arenas et al. (2018), the min-max approach is just one of the possible ways to represent and model the fairness. Alternative approaches include deprivation-like models (as reported in Sect. 3) or survival functions (see, e.g., Knight et al. 2012). Our approach is in continuity with the work Talarico et al. (2015), which inspired this work, and justified by the fact that the min-max approach seems more suitable to evaluate the price of fairness (Nicosia et al. 2017) through the hierarchical objective functions (16) and (17).

**Fig. 3:**
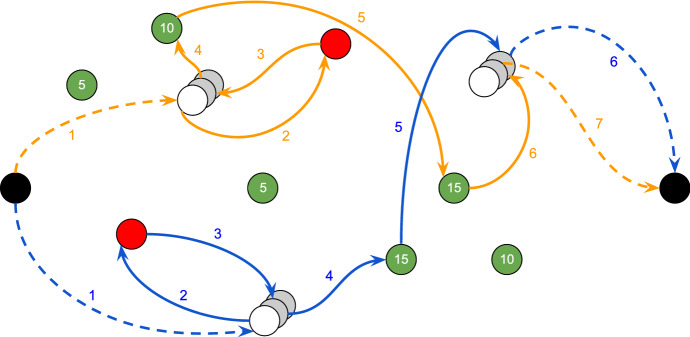
Example: fairness solution of a TOP formulation representing the ambulance tours after a disaster. Each tour starts from the starting dummy node and ends in the ending dummy node, and hospitals are always visited in accordance with the graphic overlay. Numbers on arcs indicate the order in which they are visited in the tour

To explain better how the two objective functions $z_f$ (16) and $z_e$ (17) work, we can consider the example depicted in Fig. 1, which could represent the best solution for the proposed model using $z_e$. We can observe that in the orange tour a red patient is visited after a green patient. Such a tour can not be an optimal solution using $z_f$: as a matter of fact, a change of the visit order of those patients can minimise the value of $C_{max}$, which means that red patients have a lower maximum completion time. Generally, we expect that setting the fairness component at the higher level of the hierarchical objective function, red patients are all visited at the beginning of the |*K*| tours, as depicted in Fig. 3. After minimising the value of $C_{max}$, the objective function (16) maximises the scores collected in the green nodes without having an impact on the red patients. Conversely, the tours in Fig. 1 maximises the collected scores, but they take into account how soon the red patients are visited, unless it is possible to decrease their completion time without decreasing the value of $\sigma _G$.

## Solution algorithm

As soon as the complexity of the instance increases due to an increase in the number of patients and/or the number of teams, an *ad hoc* and efficient solution algorithm is required. In this section, we report a new hybrid algorithm based on a machine learning and neighbourhood search, which is capable of largely reducing the solution running time especially when the complexity of the problem increases. In the following, we report the basic elements of the algorithm highlighting its differences when dealing with the two different objective functions $z_f$ and $z_e$. Finally, we describe the whole solution algorithm.

### Initial solution based on a machine learning approach

Due to the complexity of computing a tour for each ambulance (as depicted in Figs. 1 and 3), the basic idea is to find proper clusters of nodes belonging to *N* to ease the computation of good initial tours. This approach takes up the well-known *cluster-first route-second* algorithm for solving routing problems with some *ad hoc* improvements.

The clusters of nodes belonging to *N* are computed through a Machine Learning algorithm called Spectral Clustering (SC) (Ng et al. 2001). The spectral clustering is an algorithm used to identify communities of nodes in a graph based on the edges connecting them. It makes use of a similarity matrix that consists in a quantitative assessment of the relative similarity of each pair of nodes in the graph or points in the dataset. In our implementation, the similarity matrix is a function of the distance between two nodes in such a way that closer nodes are more similar than distant nodes. The advantage of using a spectral clustering algorithm instead of the classical K-Means algorithm (MacQueen 1967) is to have a more general and effective initial procedure, which can be adapted to different classes of instances having different distance measures.

In the beginning, the set *G* is clustered in |*K*| clusters using the SC algorithm. Then, the cardinality of the clusters obtained from *G* is balanced by moving a green node from a cluster to another in such a way to minimise the average distance of the destination cluster to the others. After computing some clusters from the set *R* using the SC algorithm, each red cluster is added to the closest (in terms of average distance among nodes) green cluster. Again, to regain a balanced cardinality, red nodes are moved to other clusters to minimise the average distance of the destination cluster to the others. Finally, the nodes belonging to *H* are added to the current clusters following the same procedure adopted for the nodes in *R*.

Such clusters are then the input for a single team customised version of the linear program described in Sect. 3 to compute the initial tour of the team associated with each one of the |*K*| clusters. When dealing with the $z_f$ objective function, the customised single team linear program imposes that all red nodes are visited before the first green node. Conversely, when dealing with the $z_e$ objective function, we adopt the same customised linear program but with the aim of maximising the overall score.

### Improving the solution using ad hoc neighbourhoods

The initial solution is composed of |*K*| feasible tours that could be not optimal. Such a solution is then improved by a neighbourhood search. First, we describe the ad hoc neighbourhoods and then how they are used within the neighbourhood search framework.

*Neighbourhoods for red patients.*

We introduce three neighbourhoods aimed at intensifying or diversifying the search on the current solution operating on nodes belonging to *R*.

The neighbourhood $N_1$ swaps two red nodes belonging to the same tour to generate a new feasible tour aimed at reducing the overall tour duration. On the contrary, the neighbourhood $N_2$ swaps two red nodes belonging to two different tours to generate two new feasible tours aimed at reducing the duration of each tour. Finally, neighbourhood $N_3$ shifts one red node (and the subsequent hospital node) from a tour to another one in such a way to generate a feasible solution aimed at reducing the duration of each tour.

All the neighbourhoods select the best feasible move (best improvement) but only $N_2$ and $N_3$ select such a move even if it worsens the current one. To avoid cycles, tabu lists have been introduced as depicted below.

*Neighbourhoods for hospitals.*

We introduce two neighbourhoods aimed at intensifying or diversifying the search on the current solution operating on nodes belonging to *H*.

The neighbourhood $N_4$ swaps one hospital node belonging to a tour with one hospital node not visited in such a way to reduce the tour duration but maintaining the feasibility. The neighbourhood $N_5$ randomly selects one-third of the nodes in *H* and visited by one tour. Each selected node is then substituted by another node in *H* but not visited.

Both neighbourhoods select the best feasible move (best improvement) even if it worsens the current one. To avoid cycles, tabu lists have been introduced as depicted below.

*Neighbourhoods for green patients.*

We introduce three neighbourhoods aimed at intensifying or diversifying the search on the current solution operating on nodes belonging to *G*.

The neighbourhoods $N_6$ and $N_7$ are aimed at improving the overall priority score. The neighbourhood $N_6$ swaps one green node belonging to a tour with one green node not visited in such a way to generate a feasible solution inserting the new node to reduce, if possible, the tour duration and, in the case of $z_f$, inserting the new green node after all the reds. The neighbourhood $N_7$ inserts one green node not visited into a tour in such a way to generate a feasible solution inserting the new node to reduce, if possible, the tour duration and, in the case of $z_f$, inserting the new green node after all the reds.

All the neighbourhoods select the best feasible move (best improvement) even if it worsens the current one. To avoid cycles tabu lists have been introduced as depicted below.

Finally, the neighbourhoods $N_8$ removes one-third of the nodes belonging to *G* and visited by one tour selecting them among those with a minimal value of the ratio

$$\begin{aligned} \dfrac{s_i}{t_{hi} + f_i + t_{ij}} \end{aligned}$$

where *h* and *j* are respectively the node that precedes and that follows the node *i*. The rationale is to remove from the solution those patients whose marginal score is minimum to free up time in such a way to allow the visit of more *profitable* patients. Note that the ratio is updated after each deletion.

*Avoiding cycles.*

To avoid cycles during the exploration of the above neighbourhoods, two types of tabu lists are used.

The first one $L_1$ avoids a node to come back to its starting tour for a number of iterations after a move while the second one $L_2$ blocks one node to be moved from its destination tour for a number of iterations after a move. According to this description and the type of nodes, we use $L^G_1$ and $L^G_2$ (with lengths $\ell ^G_1$ and $\ell ^G_2$, respectively) for green nodes, $L^H_1$ and $L^H_2$ (with lengths $\ell ^H_1$ and $\ell ^H_2$, respectively) for hospital nodes, and $L^R_1$ and $L^R_2$ (with lengths $\ell ^R_1$ and $\ell ^R_2$, respectively) for red nodes. All four tabu lists are implemented using tabu tags (Gendreau et al. 1994).

*The solution algorithm.*

We have all the elements to describe the complete algorithm. A pseudocode of our solution approach is reported in Algorithm 1.

After computing the initial solution (line 1), the algorithm starts a cycle of $I_{tot}$ iterations (line 4). At each iteration, the selected neighbourhood is visited once generating a new solution (lines 5 and  6). Such a neighbourhood is selected from the sequence $N_1$, $N_2$, $N_4$, $N_6$, $N_7$ and $N_3$: for instance, after using $N_1$ the next iteration uses $N_2$, and the next $N_4$, and so on. After $I_{ni}$ not improving iterations (line 11), the algorithm restarts the search applying the neighbourhoods $N_5$ and $N_8$. The algorithm ends by returning the best solution computed (line 12).
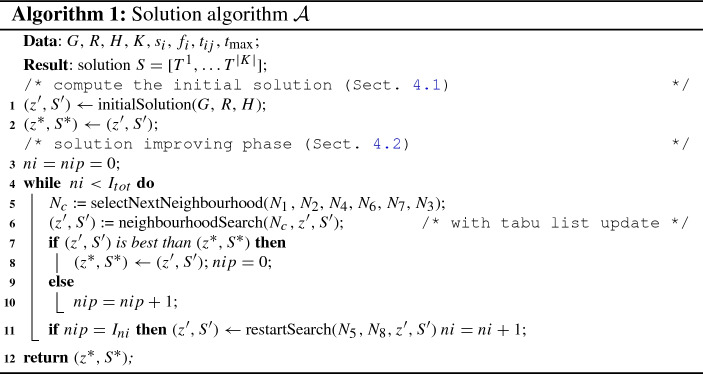


## Quantitative analysis

In this section we present a quantitative analysis performed on a set of realistic instances (reported in Sect. 5.1) in order (i) to evaluate the quality and the efficiency of the algorithm ${\mathcal {A}}$ proposed in Sect. 4, and (ii) to compare the fair solution and the system optimum (reported in Sect. 5.2).

### The generation of realistic instances

We generate a set of 24 benchmark instances exploiting the instance generator presented in Aringhieri et al. (2019), which has been modified in such a way to partition the nodes into three different types (hospital, green patient and red patient) and to label: (i) the green patients with a score in 5, 10, 15 with uniform probability, (ii) all patients with a service time (minutes) generated with a uniform distribution in [5, 35] for the green patients and in [2, 15] for the red patients, (iii) all hospitals with a fixed service time of 10 minutes. Coordinates have been scaled considering a square area of $900 \, km^2$, then travelling times have been computed through the Euclidean distance between nodes and considering an average speed of 50 km/h.

We generated 8 instances for three different numbers of patients: 10, 25 and 50. The size of the instances is the same of those tested in Talarico et al. (2015). Each instance differs for the coordinates of the nodes, the distribution of the red patients (scattered or clustered), the number of hospitals, and the number of ambulances. Then, for each instance, we used the 1-tree bound for the Travelling Salesman Problem (Valenzuela and Jones 1997) as lower-bound of the travelling time needed to visit all the nodes with only 1 ambulance. We sum all the service times to the 1-tree bound value to have a lower bound *LB* of the total time. Then, we find a value of in $[ 0.8 \, LB, 1.2 \, LB ]$, in such a way to have feasible but non-trivial solutions (e.g., the available time is sufficient to serve all the red patients but not large enough to visit all the green ones easily). The characteristics of the instances are resumed in Table 2, in which we highlight how the dimension of the graph increases with the increasing of the number of ambulances, hospitals and red patients, because of the number of dummy hospital nodes, that is $|D| = |K| + |O| \,( |K| + |R| )$.

**Table 2:** Characteristics of the realistic benchmark instances

id	|*N*|	|*G*|	|*R*|	Red distr.	|*O*|	|*D*|	|*K*|	$T_{\max }$ ($\min$)
P10C3O1	18	7	3	Clustered	1	5	1	248
P10S3O1	18	7	3	Scattered	1	5	1	248
P10C4O1	19	6	4	Clustered	1	6	1	315
P10S4O1	19	6	4	Scattered	1	6	1	315
P10C3O2	26	7	3	Clustered	2	12	2	135
P10S3O2	26	7	3	Scattered	2	12	2	135
P10C4O2	28	6	4	Clustered	2	14	2	135
P10S4O2	28	6	4	Scattered	2	14	2	135
P25C6O2	54	17	8	Clustered	2	25	3	203
P25S6O2	54	17	8	Scattered	2	25	3	203
P25C8O2	58	15	10	Clustered	2	29	3	225
P25S8O2	58	15	10	Scattered	2	29	3	225
P25C6O3	70	17	8	Clustered	3	40	4	167
P25S6O3	70	17	8	Scattered	3	40	4	167
P25C8O3	76	15	10	Clustered	3	46	4	169
P25S8O3	76	15	10	Scattered	3	46	4	160
P50C12O3	120	35	15	Clustered	3	65	5	203
P50S12O3	120	35	15	Scattered	3	65	5	180
P50C16O3	135	30	20	Clustered	3	80	5	248
P50S16O3	135	30	20	Scattered	3	80	5	257
P50C12O4	146	35	15	Clustered	4	90	6	158
P50S12O4	146	35	15	Scattered	4	90	6	158
P50C16O4	166	30	20	Clustered	4	110	6	225
P50S16O4	166	30	20	Scattered	4	110	6	212

### Computational analysis

The aim of this section is to report the quantitative analysis on the realistic instances described in the previous section. First we summarise the analysis performed using a general purpose solver to solve the mathematical model with the hierarchical objective functions $z_f$ (16) and $z_e$ (17). Then, we discuss the efficiency and the quality of the solutions computed by our algorithm. Finally, we provide a comparison between the $z_f$ and $z_e$ solutions to assess the so called price of fairness (Nicosia et al. 2017).

All the computational tests have been performed on a standard desktop computers equipped with a Intel Core i7-8700 3.20GHz with 12 cores, and 16 Gb of memory. The integer linear programs resulting from the discussion reported in Sect. 3 has been implemented adopting OPL language and solved with CPLEX 12.9 with default settings. We would remark that the default settings use all the cores available reducing the overall running time instead of using only one core. The algorithm ${\mathcal {A}}$ has been implemented in Python using the following parameter settings: $I_{tot} = 2000$, $I_{ni} = 20$, $\ell ^G_1 = 5$ and $\ell ^G_2 = 7$, $\ell ^H_1 = 5$ and $\ell ^H_2 = 7$, $\ell ^R_1 = 10$ and $\ell ^R_2 = 15$.

Table 3 reports the computational results obtained with CPLEX on the first 8 instances, for which the optimal solution has been computed. The table contains two sets of columns. The first set (under the name *fairness*) reports the values $z_f$ and $C_{\max }$ of the solution computed, and the running time in seconds; then the columns $z_e$ and $\sigma _G$ report the efficiency values computed on the fairness solution. On the contrary, the second set (under the name *efficiency*) reports the values $z_e$ and $\sigma _G$ of the solution computed, and the running time in seconds; then the columns $z_f$ and $C_{\max }$ report the fairness values computed on the efficiency solution. The columns have the same meaning also in Tables 4 and 5 .

**Table 3:** CPLEX: computational results over the benchmark instances with 10 patients

	*Fairness*					*Efficiency*				
	$z_f$	$C_{\max }$	secs.	$z_e$	$\sigma _G$	$z_e$	$\sigma _G$	secs.	$z_f$	$C_{\max }$
P10C3O1	11,987	182	7.5	6043	25	10,959	45	0.4	16,191	246
P10S3O1	7698	138	19.8	7332	30	10,961	45	0.3	13,619	244
P10C4O1	15,509	219	96.7	12,421	40	17,077	55	0.2	21,458	303
P10S4O1	14,676	241	11.4	7659	25	12,335	40	0.2	18,565	305
P10C3O2	5783	88	294.9	3312	25	6679	50	15.9	7936	121
P10S3O2	4063	73	383.5	3327	25	5986	45	13.5	7459	134
P10C4O2	6218	88	667.2	3992	30	7355	55	12.9	8820	125
P10S4O2	6659	94	229.4	1946	15	7353	55	18.8	8962	127

We observe a consistent difference in running time: actually, the computation of a fairness solution requires ten times the running time needed for computing an efficient solution. We observe similar running time differences for the larger instances reported in Table 2. Furthermore, several hours are required for bigger instances often to find a sub-optimal solution. Since the aim of our problem is to provide a prompt decision in a situation of post-disaster management, this fact highlights the need for the solution algorithm presented in Sect. 4.

**Table 4:** Algorithm ${\mathcal {A}}$: computational results over the benchmark instances with 10 patients

	*Fairness*					*Efficiency*				
	$z_f$	$C_{\max }$	secs.	$z_e$	$\sigma _G$	$z_e$	$\sigma _G$	secs.	$z_f$	$C_{\max }$
P10C3O1	**11,987**	182	3.8	6043	25	**10,959**	45	4.0	16191	246
P10S3O1	**7698**	138	4.0	7332	30	**10,961**	45	4.1	13,619	244
P10C4O1	**15,509**	219	4.7	12,421	40	**17,077**	55	4.9	21,458	303
P10S4O1	**14,676**	241	4.4	7659	25	**12,335**	40	4.6	18,565	305
P10C3O2	6235	95	6.0	4665	35	**6679**	50	5.1	7936	121
P10S3O2	**4063**	73	6.0	3327	25	**5986**	45	4.3	7459	134
P10C4O2	**6218**	88	5.0	3992	30	**7355**	55	4.2	8820	125
P10S4O2	7567	107	5.2	3973	30	**7353**	55	4.7	8962	127

**Table 5:** Algorithm ${\mathcal {A}}$: results over the benchmark instances with 25 and 50 patients

	*Fairness*					*Efficiency*				
	$z_f$	$C_{\max }$	secs.	$z_e$	$\sigma _G$	$z_e$	$\sigma _G$	secs.	$z_f$	$C_{\max }$
P25C6O2	22,413	128	21.6	23,217	115	26,191	130	21.2	34,894	199
P25S6O2	26,488	143	23.7	22,187	110	28,223	140	25.7	36,502	197
P25C8O2	25,657	152	28.2	21,852	95	28,654	140	24.4	35,435	201
P25S8O2	26,264	154	32.3	14,756	70	27,483	130	25.1	35,267	207
P25C6O3	16,620	95	28.5	16,205	100	23,474	145	25.9	28,191	161
P25S6O3	15,514	84	26.9	17,846	110	25,103	155	26.6	29,977	162
P25C8O3	20,025	125	39.7	16,875	100	23,632	140	34.2	26,908	168
P25S8O3	15,025	100	37.7	11,975	75	22,380	140	29.3	24,020	160
P50C12O3	56,464	159	102.7	28,261	140	43,444	215	88.7	71,341	201
P50S12O3	42,570	120	103.3	27,030	150	40,546	225	88.4	63,499	179
P50C16O3	55,254	184	186.5	30,366	130	47,941	205	133.6	70,229	234
P50S16O3	65,043	213	181.4	34,482	135	56,285	220	146.6	77,810	255
P50C12O4	53,200	150	121.2	36,050	200	38,735	215	105.3	63,865	180
P50S12O4	43,267	122	138.0	26,113	165	43,567	275	108.5	55,973	158
P50C16O4	61,582	202	197.8	58,908	230	69,135	270	159.5	77,760	255
P50S16O4	46,761	156	214.9	41,379	195	56,234	265	176.7	63,246	211

Table 4 reports the computational results of the proposed solution algorithm where the numbers in bold highlight the optimal solutions with respect to the optimal values reported in Table 3. From a comparison with the values reported in Table 3, the results in Table 4 prove the quality of the solution computed by the algorithm ${\mathcal {A}}$: actually, the algorithm *A* replicates all the optimal values in the efficiency case, and 6 over 8 in the fairness case. Furthermore, the results prove the efficiency of the proposed algorithm, which is capable of obtaining such results in a few seconds instead of tens (efficiency) or hundreds (fairness) of seconds.

In order to evaluate the impact of each neighbourhood on the quality of the final solutions $z_f$ and $z_e$, we solved the ten instances reported in Table 3 temporarily disabling one of the eight neighbourhoods resulting in 160 new solutions, which are then compared with the results reported in Table 4.

When dealing with the fairness, we observed significant variations in $z_f$ (i.e., at least $0.5\%$) only for $N_2$ and $N_6$. We also consider the value of $z_e$ computed on the fairness solution. Without $N_2$, the value of $z_f$ worsens of the $6.02\%$ while no significant variations are reported in the value of $z_e$. On the contrary, no significant variation of $z_f$ are reported without $N_6$ while the value of $z_e$ worsens of the $3.29\%$.

When dealing with the efficiency, we observed no significant variations in $z_e$ (i.e., at least $0.5\%$) temporarily disabling one of the eight neighbourhoods. On the contrary we reported a significant worsening of the value of $z_f$ computed on the efficiency solution ranging from $0.62\%$ to $0.98\%$.

We performed a further test temporarily disabling all the neighbourhoods working on a specific component of the solution, that is (i) the neighbourhoods for red patients $N_1$, $N_2$ and $N_3$, (ii) the neighbourhoods for hospitals $N_4$ and $N_5$, and (iii) the neighbourhoods for green patients $N_6$, $N_7$ and $N_8$. The results clearly get worse in all the tests. We observed a peak of $6.31\%$ for $z_f$ when dealing with the fairness and disabling $N_1$, $N_2$ and $N_3$. On the contrary, we observed a peak of $10.58\%$ for $z_e$ when dealing with the efficiency and disabling $N_6$, $N_7$ and $N_8$.

Table 5 reports the computational results of the algorithm over the remaining benchmark instances, that is those with 25 and 50 patients. The running time of the algorithm proves its capability of dealing with larger and more realistic instances. Fairness solutions seem harder to be computed than the efficiency one, at least in terms of running time.

A preliminary comparison between the two kinds of solutions can be done as follows. Moving from an efficient solution to a fair one, it is possible to measure the benefit in terms of fairness gained and efficiency lost. Vice versa, we can measure the benefit in terms of efficiency gained and fairness lost. In the former case, the average fairness gained is about $27.71\%$ while the efficiency lost is about $29.87\%$. In the latter case, the average efficiency gained is about $46.10\%$ while the fairness lost is about $40.63\%$. These results seem to prove that there is no dominance between the two kinds of solutions.

**Fig. 4:**
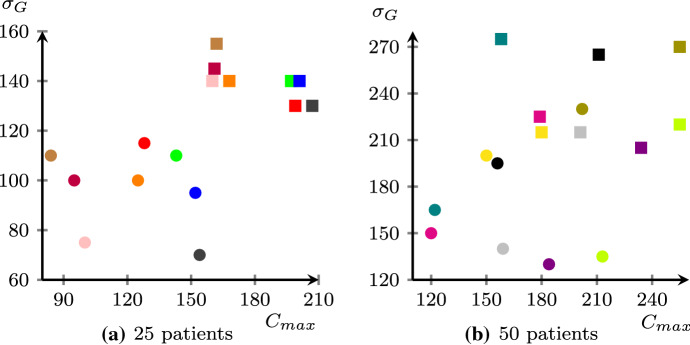
Comparing the solution provided by the algorithm ${\mathcal {A}}$ using $z_f$ (circles) and $z_e$ (squares) as objective functions (same color is used for the two solutions of the same instance)

No significant difference emerges from dealing with instances with a different distribution of the red patients over the disaster area: similar completion times have been obtained for such patients on the clustered and scattered version of the same instances. For example, P25C6O2 has a slightly lower value of $C_{max}$ with respect to P25S6O2, but the opposite occurs comparing P25C6O3 and P25S6O3. In order to better appreciate the trade off between $C_{max}$ and $\sigma _G$ provided by the two different objective functions $z_f$ and $z_e$, we plot the solution obtained for the instances with 25 and 50 patients in Fig. 4. Both fairness and efficiency solutions provided for the same instances are far from dominating each other. Furthermore, smaller instances show a clear separation between fairness and efficiency solutions, regardless of how patients are distributed over the disaster area. Such a separation vanishes as the number of patients to be visited increases, but without loosening the strong trade off between the two objectives.

From the reported analysis that can be seen as a sort of Pareto analysis, it is evident that no clear dominance between the fairness and efficiency solutions has been identified. From an application perspective, this empirical result makes clearer the meaning of *price of fairness* (Nicosia et al. 2017): actually, to improve the efficiency of a fairness solution, it is necessary to allocate more time resources to serve the same green patients served by the efficient solution.

## Conclusions

Although many researchers concerning humanitarian relief services highlight the importance of equity, the few articles focused on fairness as an objective in disaster optimisation problems as highlighted in our literature review. As discussed in Nicosia et al. (2017), modelling fairness is in itself challenging since the concept of fairness can vary in accordance with the context of the problem. This results in modelling approaches determining solutions with different quality. Regarding relief operations, the concept of fairness can be defined as equity and impartiality at the service level for people who are in need and, by consequence, the management of the operations in the immediate aftermath of a disaster must be done in such a way to to rescue affected people with different priority in accordance with the restrictions.

In this paper we considered the problem of finding the best ambulance tours to transport the patients in relief operations while considering fairness and equity to deliver services to patients. We formulated such a problem as a new variant of the Team Orienteering Problem with hierarchical objectives to address also the efficiency issue. Since the aim of our problem is to provide a prompt decision in a situation of post-disaster management, we developed a new hybrid algorithm based on a machine learning and neighbourhood search capable of largely reducing the solution running time especially when the complexity of the problem increases. Our computational tests are based on a new set of realistic benchmark instances. Finally, the comparison between the two objectives showed a clear absence of dominance between fairness and efficiency.

Fairness is one of the central arguments of our work. As reported this study provides initial but interesting insights that should be further investigated. From this point of view, it could be of interest to test different approaches to deal with fairness in accordance with the remarks in Anaya-Arenas et al. (2018), Huang et al. (2012) and Nicosia et al. (2017). This can be further extended considering deprivation-like models (Zhu et al. 2019) or survival functions (Knight et al. 2012) as objectives.

From an application perspective, the problem can be extended considering more than two classes of urgency, hospitals with a maximum capacity in terms of patients to be served, and to explicitly consider a multi level hierarchy of hospitals. Other extension could consider a heterogeneous fleet of emergency vehicles, which means that only a subset of vehicles can serve some particular patients. We considered a static version of the problem in which all the patients are known in advance. Our work can be extended to fit a more realistic situation in which new patients might arrive over time. A possible solution is to use our algorithm within a more general online optimisation framework, as discussed in Aringhieri (2019).

From a methodological perspective, this new variant of the team orienteering problem deserves a detailed analysis of the its characteristics, and a deep study of the problem in order to provide general solution algorithms as discussed in Aringhieri et al. (2021). It could be of interest to study a different and more flexible way to consider the maximum time limit for the duration of a tour: instead of having a strong constraint on time limit as the depicted by Eq. (7), the mathematical model discussed in Sect. 3 can be modified adding a sort of penalisation of the total duration time of an ambulance. Finally, a possible improvement of the algorithm proposed in Sect. 4 is that of considering nested neighbourhoods within an Adaptive Neighbourhood Search framework.
